# A Chemical Test of the Antiquity of Bones

**Published:** 1893-08

**Authors:** 


### SELECTIONS.

### A Chemical Test of the Antiquity of Bones.

The effort was made by M. Adolphe Carnot last summer, in a paper read before the Academie des Sciences, Paris, to establish a chemical measure of the antiquity of bones. He claimed that this is shown by the amount of fluorine they contain. Its relative proportion increases as the bones are older. Representing the maximum by 1, modern bones show but .06, those from the old quaternary strata .35, those from the tertiary .64. Hence, when human and other bones are found in the same strata, and the question whether they should be assigned to the same age arises, analysis is claimed to offer a solution; and M. Emile Riviere had recourse to it as the crucial test in the disputed age of some human bones found along with those of extinct species in the gravels near Billancourt, on the Seine; proving, he believed, that the human bones were intrusive and late.

It seems to me, however, that this test, which I learn about from an abstract in the Journal de l'Alliance Scientifique, March 15, is open to some serious risks.

Not only do the inorganic constituents of bone differ largely in the different osseous tissues of the same skeleton, but they notoriously vary greatly at the different epochs of life. According to the analysis of Heintz, the fluoride of calcium in the average femur of an adult is about 3.5 of its inorganic constituents. Where the proportion in ancient bones differs notably from that in modern, how can we decide what part of it is owing to post-mortem changes conditioned on the quality of the soil, the amount of percolation, the length of exposure before inhumation, and the like incidents? While it would be most desirable to have at hand a positive chemical test of antiquity, we must hesitate to accept as conclusive one which seems exposed to be influenced by these precarious conditions.

